# Analysis of genetic variants for different horn phenotypes and their inheritance in Icelandic sheep

**DOI:** 10.5194/aab-67-237-2024

**Published:** 2024-06-05

**Authors:** Rebecca Simon, Karólína Elísabetardóttir, Gesine Lühken

**Affiliations:** 1 Institute of Animal Breeding and Genetics, Justus Liebig University, Gießen 35390, Germany; 2 Hvammshlíð, Blönduós, 541, Iceland

## Abstract

Icelandic sheep are characterized by a great diversity in horn phenotypes. Within their breed, they show a variability in terms of this trait to an extent rarely observed elsewhere. Previously, several genetic variants were published as markers for horn status (in terms of absence or presence of horns, including scurs) and horn traits (e.g., oval horns, horn length and polyceraty). The aim of this study was to genotype, for the first time, five of these genetic variants in Icelandic sheep with different horn phenotypes, as well as to analyze their inheritance. Phenotypic and pedigree data, as well as DNA samples from two Icelandic sheep farms, were used. Genetic variants were genotyped by published PCR-based methods in all samples ( n=94 ) or in subsets. As in other sheep breeds with variable horn status, the inheritance of the presence or absence of horns was shown to be complex in Icelandic sheep, especially when sheep carry anything other than regularly formed horns. The 1.78 kb sized *RXFP2* insertion on ovine chromosome 10 previously described to be associated with polledness in several sheep breeds was also found to be present in Icelandic sheep and showed some association but not a perfect segregation with the individuals' horn statuses. Missing associations were especially seen in sheep with scurs and oval horns. Regarding horn shape, there was no agreement with the studied variants described in Chinese breeds having comparable horn traits. However, matching tendencies were seen for the horn size variant that was found in the same study. All sheep with four or more horns carried the already published 4 bp deletion in *HOXD1*, as previously described for three other sheep breeds. Interestingly, for the first time, the deletion was also detected in phenotypically polled animals originating from multi-horned families. According to the results from animals genotyped simultaneously for the *RXFP2* and the *HOXD1* variants, polledness in sheep with a genetic disposition for polyceraty seems not to be controlled by the *RXFP2* insertion. However, this and all other findings in Icelandic sheep need to be confirmed by analyzing a higher number of well-phenotyped animals.

## 1 Introduction

**Figure 1:**
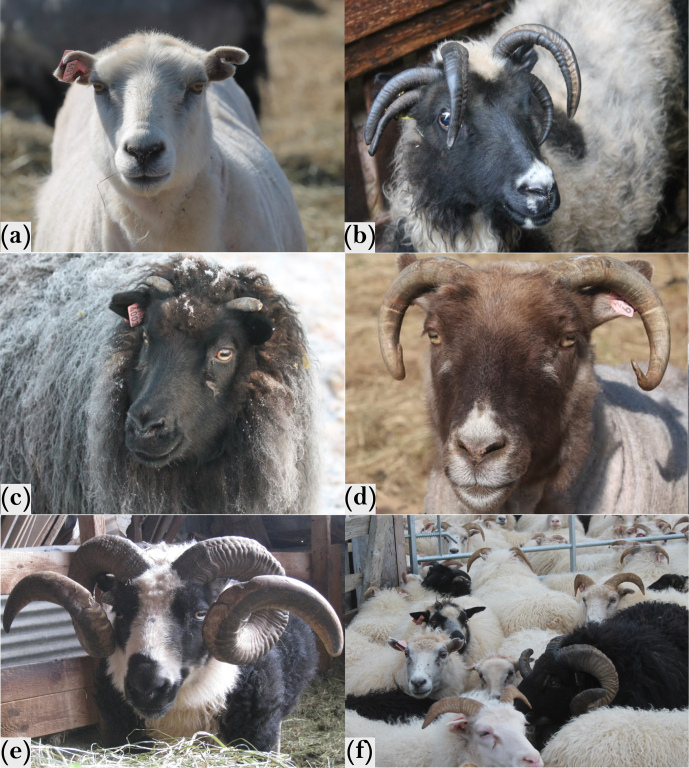
An example for the diversity of the horn status in Icelandic sheep of both sexes. **(a)** Polled mutton. **(b)** Polycerated ewe (six horns). **(c)** Ewe with scurs (horn-like structures). **(d)** and **(e)** Different horn shapes in rams: oval **(d)** and normal “spiral” horns **(e)**. **(f)** A flock with polled and horned individuals.

Iceland, due to its isolated island location and strict import restrictions for animals, is a particularly interesting area for research. One example of an interesting research object is the northern European short-tailed Icelandic sheep (short: Icelandic sheep), which were originally formed by various northern European breeds brought to the island by the Viking settlers between 800 and 1000 AC (Dýrmundsson and Niżnikowski, 2010). It is the only existing sheep breed in Iceland today and has not been crossed with foreign breeds for centuries (Eythorsdottir et al., 2008; Dýrmundsson and Niżnikowski, 2010). A recent diversity study shows that the genetic influence of foreign breeds imported only occasionally in the past is negligible for the recent Icelandic sheep (data not shown, publication in preparation). To some extent, this is comparable to the much-studied population of feral Soay sheep in the archipelago of St. Kilda, Scotland (Clutton-Brock and Pemberton, 2009). Nevertheless, the Icelandic sheep show a great phenotypic variability with respect to different traits (Porter et al., 2016). A striking characteristic is the horn phenotype, which seems to be polymorphic in males and females (Fig. 1).

In the inheritance of horns or polledness, the *RXFP2* gene on ovine chromosome 10 plays a major role (Wiedemar and Drögemüller, 2015; Pickering et al., 2009), although it has already been shown for some breeds with variable or sex-linked horn status that the published 1.8 kb insertion in the 3'-UTR region of this gene is not associated with polledness (Lühken et al., 2016; He et al., 2016). Duijvesteijn et al. (2018) succeeded in the genomic prediction of the presence or absence of horns in Merino sheep using two highly significant single nucleotide variants (SNVs) on ovine chromosome 10 (OAR10_29458450 and OAR10_29546872.1) as markers. Evidence of one of the two is already considered to be sufficient for the prediction, but this has only been proven in Merino sheep (Duijvesteijn et al., 2018). A total of 68 genes were identified recently that show a down- ( n=10 ) or up-regulation ( n=58 ) during horn bud development in sheep embryonic development (Luan et al., 2023). Luan et al. (2023) state that the results of the expression analyses indicate that only a few genes are involved in horn development – including the often-mentioned *RXFP2*.

In addition to polled (“kollótt”, Fig. 1a; scured, Fig. 1b–c), and horned (“hyrnt”, Fig. 1d–e) individuals, there are also Icelandic sheep that carry a multitude of horns (four to six horns, polyceraty) (Dýrmundsson, 2005). Interestingly, those can also be polled or scured. Breeders are able to differentiate between polled sheep of two-horned origin and of polycerate origin based on the shape of the skull.

The cause for the evolution and persistence of the polyceraty trait has not yet been explained. It is assumed that the emergence of supernumerary horns is the result of a split in the horn buds during embryo development (Allais-Bonnet et al., 2021). The dominant trait of polyceraty in sheep was recently shown to be associated with a short deletion (4 bp sized) in the *HOXD1* gene (Allais-Bonnet et al., 2021) after it had been mapped on ovine chromosome 2 previously, which was confirmed by GWAS for Damara, Jacob, and Navajo-Churro sheep (Kijas et al., 2016; Greyvenstein et al., 2016). The association with a region on chromosome 2 was confirmed for three Chinese breeds as well (He et al., 2016; Ren et al., 2016).

In addition to the presence or absence and/or the number, the shape and size of the horns can vary in Icelandic sheep as well. One can find oval horns that do not have sharp edges in cross-section but also normal “spiral” ones in both sexes (Fig. 1d–f). The same region in which the *RXFP2* gene is located was found to be associated with the horn type and base circumference in male Soay sheep (Johnston et al., 2010). In the same region, a quantitative trait locus (QTL) for the dimension of horns has been found in bighorn sheep, *Ovis canadensis* (Kardos et al., 2015; Poissant et al., 2012). A haplotype within and around the *RXFP2* gene, specifically one SNV (OAR10_29461968) of this haplotype, was shown to segregate with horn length, as well as with horn shape, in an investigation with different Chinese sheep breeds (Pan et al., 2018).

The aim of this study was to analyze, for the first time, the previously mentioned genetic variants known or suspected to influence different horn phenotypes (Table 1), as well as the inheritance of horn phenotypes in Icelandic sheep.

**Table 1:** Overview of previously published variants used for genotyping of horn-related phenotypes in Icelandic sheep.

Name position	Gene	Ovine chromosome	Associated with	Breed(s)	Reference
1.78 kb in-sertion g.29 456 048–29 457 880 (OARv3.1)	*RXFP2*	10	Polledness	Bündner Oberländer sheep, Valais Red sheep, Valais Blacknose sheep, Engadine Red sheep, Swiss Black-Brown Mountain sheep, Swiss Mirror sheep, Swiss White Alpine sheep	Wiedemar and Drögemüller (2015)
OAR10_29458450 (TT)	Close to *RXFP2*	10	Polledness	Merino sheep	Duijvesteijn et al. (2018)
OAR10_29461968 (TT)	*RXFP2*	10	Increased horn length	Oula sheep, Prairie Tibetan sheep, Valley Tibetan sheep, Small Tail Han sheep	Pan et al. (2018)
OAR10_29461968-OAR10_29462010 (TT,“haplotype 2”)	*RXFP2*	10	Horn shape → curled rather than oval	Oula sheep, Prairie Tibetan sheep, Valley Tibetan sheep, Small Tail Han sheep	Pan et al. (2018)
4 bp deletion(AGTA/–) g.132,832,249-132,832,252del (Oar_v4.0 assembly)	*HOXD1*	2	Polyceraty	Jacob sheep, Navajo-Churro sheep, Damara sheep	Allais-Bonnet et al. (2021)

## 2 Material and methods

### 2.1 Animals

In total, samples from 94 Icelandic sheep were collected. Samples from 61 sheep (26 males and 35 females) originated from a single farm in Iceland (no. 1). Furthermore, pedigree information from nine additional sheep was used, but no DNA samples were available. As, in that specific farm, no polycerate sheep were available, we additionally received samples from 33 sheep from another Icelandic farm with a known presence of polycerate sheep (no. 2). This sample set contained both multi-horned (four to six horns) and normally horned sheep, as well as polled ones belonging to those two groups. Further detailed information on the animals used for the analyses can be found in Table 2.

**Table 2:** Overview of sheep samples used according to general and, where necessary, detailed horn status, as well as sex.

Farm	General horn status	n	Sex	n	Details on horns	Sex	n
No. 1	Polled (kollótt)	31	Female	26			
			Male	6			
	Horned (hyrnt)	21	Female	3	Oval (sívalhyrnt)	Female	1
			Male	18		Male	11
					Normal (hyrnt)	Female	2
						Male	7
	Scurs (smáhnýflótt)	9	Female	6			
			Male	3			
No. 2	Polyceraty	33	Female	13	Four to six horns	Female	2
			Male	20		Male	2
			Unknown	1	Polled (four to six horns)	Female	2
						Male	1
						Unknown	1
					Two horns	Female	5
						Male	14
					Polled (two horns)	Female	4
						Male	2

Sample collection was initially performed for diagnostic purposes (scrapie eradication program), and remainders were provided to us for further use. 

### 2.2 DNA extraction

Depending on the sample type, DNA was extracted with either a blood kit or a tissue kit (Macherey Nagel, Düren, Germany) according to the manufacturer's instructions. Only the amount of used elution buffer for blood samples was lowered to 75 µ L in order to yield a higher DNA concentration.

### 2.3 Pedigree

The pedigrees for farm no. 1 were created according to the owner's information about the relatedness of the animals, supported by the herd book information. Complete pedigree information, including information about the parents' horn status, up to the third or fourth generation, was available for most of the sheep from this farm (no. 1); it was only for four male and five female sheep of the first and second generations that no horn status information was available. To demonstrate the inheritance of horn status in Icelandic sheep, focusing on polled matings and scured offspring, including the influence of the previously published *RXFP2* variant (Wiedemar und Drögemüller, 2015), two partial pedigrees were constructed with the help of QuickPed (Vigeland, 2022). For the second farm, no information on the parents was available; therefore, no pedigree was drawn.

### 2.4 Genotyping

A total of 94 out of 94 samples were genotyped for the *RXFP2* variant (1.78 kb insertion). Genotyping of the three additional variants (details can be found in Table 1) was performed for a selection of the samples. It was ensured that all horn phenotypes were represented, but the focus was on individual phenotype groups: for genotyping of the haplotype published by Pan et al. (2018), the focus was on the horned individuals, with records of their horn form, including some polycerate ones. In total, 40 individuals were genotyped for these variants. For the polledness predicting SNV in merino sheep (Duijvesteijn et al., 2018), 55 individuals were genotyped, with the focus being on polled versus horned sheep (regardless of further horn characteristics). The *HOXD1* variant published by Allais-Bonnet et al. (2021) was mainly genotyped in the sheep from the polycerate flock; however, in addition, some polled and horned sheep were analyzed for comparison. This resulted in a total of 20 individuals.

For genotyping, PCR protocols as published elsewhere (Lühken et al., 2016; Pan et al., 2018; Duijvesteijn et al., 2018; Allais-Bonnet et al., 2021) were used with slight modifications and can be found in Table S1 in the Supplement.

## 3 Results

**Figure 2:**
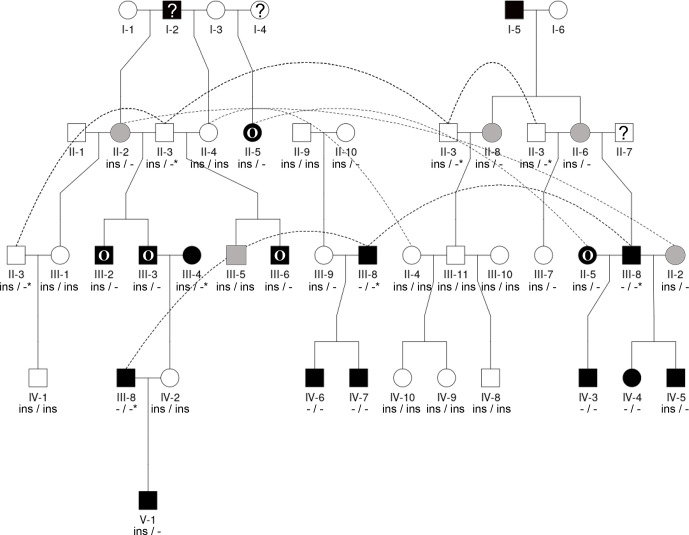
The pedigree displays the inheritance of horn status in Icelandic sheep genotyped for the *RXFP2* insertion, with the focus being on matings of polled parents. The pedigree shows five generations (I-V) including available horn status information (gray: scurs, white: polled, black: normally horned, black with O: oval horned, black with ?: horn form unknown, ?: unknown), *RXFP2* genotype ( ${}^{*}$ indicates suggested genotype), and sex (circle: female, square: male). Dashed lines indicate the same animal in different matings, e.g., the breeding ram II-3. Please note that polled matings in the Icelandic population not only result in polled offspring (IV-1, III-9, IV-8-10) but also result in oval-horned (III-6) and scured progeny (III-5).

**Figure 3:**
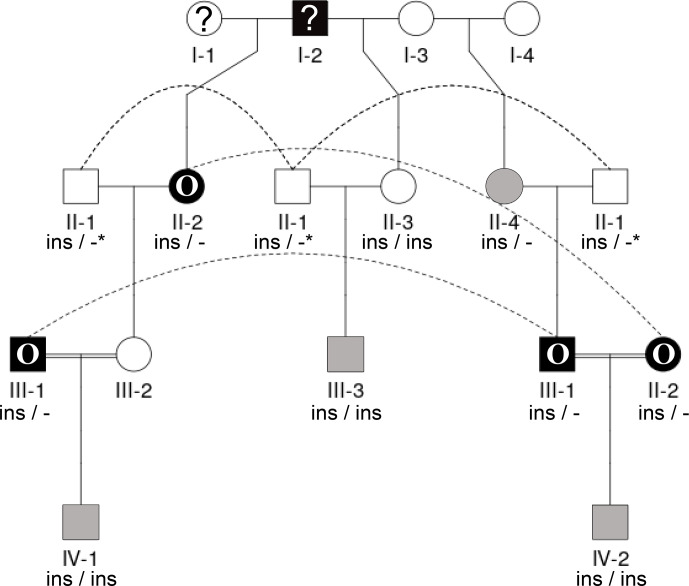
Pedigree displaying the inheritance of horn status in Icelandic sheep genotyped for the *RXFP2* insertion, starting from scured descendants in the current generation and moving backwards. The pedigree shows four generations (I-IV) including available horn status information (gray: scured, white: polled, black with O: oval horns, black with ?: horn form unknown, ?: unknown), *RXFP2* genotype ( ${}^{*}$ indicates suggested genotype), and sex (circle: female, square: male). Dashed lines indicate the same animal in different matings. III-1, III-2, and II-2 are half-siblings, indicated by a double line. Please note that animal III-2 was not available for sampling. Focusing on scured progeny, it is shown that scured males derived from polled parents (III-3), horned (oval) parents (IV-2), and a paring of a horned (oval) father and a polled mother (IV-1).

Seen as a whole, the pedigree information did not resolve the question of the inheritance mode of horn status. We found that the presence or absence of horns or scurs across the pedigree did not follow that of a simple monogenic trait. Most consistent is the very frequent occurrence of polledness among offspring from polled × polled matings (12 out of 15, Table S2). However, there are exceptions from that pattern. For example, among the six matings of polled parents displayed, four resulted in polled offspring, whereas two resulted in two male offspring with oval horns and scurs (Fig. 2) and a single male with scurs (Fig. 3).

Matings involving at least one oval-horned parent resulted in a polled female (Fig. 2), a normally horned male (Fig. 2), or even a scured male (Fig. 3) offspring. Also, a polled offspring of oval-horned parents was not observed in the sample set. A mating of two scured parents did not take place in the analyzed group of sheep. Table S2 gives a complete overview of the horn phenotype of offspring resulting from matings of parents with different combinations of their horn phenotype.

The 1.78 kb sized *RXFP2* insertion (ins) shown previously to be associated with polledness (Wiedemar and Drögemüller, 2015) was found to be present in Icelandic sheep and showed some association but not a perfect segregation with the individuals' horn statuses (Table 3). In all cases where genotyping was possible, the genotype of the offspring matches the expectation based on the genotype of the parents (Figs. 2 and 3). Except for a single polled sheep of the polycerate family, a consistent pattern is the presence of the insertion at least on one chromosome in all polled and scured sheep. In line with this, the majority of normally horned sheep (13 out of 16 males, 5 out of 6 females) did not carry the insertion at all. However, some normally horned sheep were heterozygous or homozygous for the insertion.

In contrast to normal horns, oval horns were not observed in sheep without the *RXFP2* insertion.

In sheep from polycerate families, the *RXFP2* insertion was not found to be present in the homozygous state. For four animals, the genotyping failed even after repetition. However, based on the pedigree information, it was possible to deduce the most likely *RXFP2* genotype for three animals (indicated by ${}^{*}$ in Figs. 2 and 3).

**Table 3:** Distribution of the occurrence of the 1.78 kb sized insertion in *RXFP2* depending on horn phenotype and sex of the analyzed Icelandic sheep. Please note that sheep from farm no. 2 that had no documentation of multi-hornedness were added to the respective horn phenotype group (polled or normally horned).

Horn phenotype	Sex	*RXFP2* genotype		
		(1.78 kb insertion)		
		–/–	ins/–	ins/ins
Polled (kollótt) ${}^{*}$	Female		9	18
	Male		1	7
Normally horned (hyrnt)	Female	5	1	
	Male	13	2	3
Scurs (smáhýflótt)	Female		6	
	Male			3
Oval horned (sívalhyrnt)	Female		1	
	Male		8	3
Polycerate (four to six horns) ${}^{*}$	Female	1		
	Male		1	
Polled polycerate (four to six horns)	Female	1	1	
	Male		1	
	N/A		1	

${}^{*}$ *RXFP2* genotyping failed for two additional polled (females) and polycerate sheep (one of each sex). N/A – not analyzed.

All 55 animals genotyped for the polledness predicting SNV in Merino sheep showed the wild-type allele (A); thus, there was no segregation of this variant with the examined horn status.

In the analyzed sheep, the SNV OAR10_29462010 appears to be fixed as only allele C is present. SNV OAR10_29461968 was found to be variable: C homozygotes were only found in two males with oval horns and a single female polled sheep. T homozygotes were found in all but the polled individuals tested and seem to be most frequent in normally horned animals. (Table S3).

Haplotype 2 (TT), found in Chinese breeds with curled (normal) horns, was not present in the analyzed samples, regardless of the horn phenotype. Hence, no segregation of the previously published haplotypes with a certain horn form was found.

All polycerate sheep (four- and six-horned) and five polled sheep from polycerate families were carriers of the 4 bp deletion in *HOXD1* in either a heterozygous or a homozygous state. Neither two-horned nor polled individuals from non-polycerate families (farm no. 2) carried this variant (Table 4). The same applies to the genotyped animals from farm no. 1: none carried the *HOXD1* variant.

**Table 4:** An overview of Icelandic sheep of farm no. 2 and the occurrence of the 4 bp deletion in *HOXD1* and the *RXFP2* genotype. All sheep from polycerate families (no. 1–8) carry the 4 bp deletion regardless of whether they are polycerate or polled polycerate. All remaining two-horned or polled sheep originating from farm no. 2 (No. 9–34) did not carry the *HOXD1* variant.

Sample no.	Horn phenotype	Sex	n	4 bp deletion in *HOXD1*	*RXFP2* genotype
1	Polycerate (four to six horns)	Female	1	del/–	–/–
2		Female	1	del/–	Failed
3		Male	1	del/–	ins/–
4		Male	1	del/del	Failed
5	Polled polycerate (four to six horns)	Female	1	del/del	–/–
6		Female	1	del/–	ins/–
7		Male	1	del/del	ins/–
8		N/A	1	del/del	ins/–
9–13	Horned (two horns)	Female	5	–/–	–/–
14–24		Male	10	–/–	–/–
25–26		Male	2	–/–	ins/ins
27		Male	1	–/–	ins/–
28		N/A	1	–/–	–/–
29–31	Polled (two horns)	Female	3	–/–	ins/–
32		Female	1	–/–	ins/ins
33–34		Male	2	–/–	ins/ins

N/A – not analyzed.

## 4 Discussion

Concerning Soay sheep, polledness is recessive, and males that are heterozygous in terms of the horns locus are horned, while heterozygous females carry scurs (Johnston et al., 2009). Also in the investigated families of Icelandic sheep, mating of polled parents mostly led to polled offspring, but there were few exceptions from this sign of a recessive trait. Moreover, in contrast to what had been observed in Soay sheep, scurs were not only limited to female Icelandic sheep, and this was also not a common outcome of horned × polled matings. Instead, scured males where observed in the sample set and were derived from each parental phenotype combination: both parents polled, both horned, or a horned father. These observations contradict parts of the most recent report (Johnston et al., 2009) about the mode of inheritance of polledness in sheep. Of course, for statistical approval or disapproval of any inheritance pattern, the sample set is too small, and, in some cases, the phenotypic data (horn status) of the parents were not documented. As it is possible that the mode of inheritance varies between breeds, the mode of inheritance in Icelandic sheep should be determined in a larger sample set in follow-up investigations.

As the Icelandic sheep is a breed with a variable horn status, it was expected that the 1.78 kb *RXFP2* insertion (Wiedemar and Drögemüller, 2015) would not segregate perfectly with polledness. However, in contrast to Dorper and Bovec sheep, which show a variable horn status but seem to be fixed with regard to the *RXFP2* insertion (Lühken et al., 2016), all three possible genotypes were observed in the Icelandic sheep. Most of the polled Icelandic sheep are homozygous with regard to the insertion, whereas the vast majority of horned individuals are homozygous with regard to the wild type, thus fitting more or less to what was observed for the *RXFP2* variation in uniformly horned or polled breeds (Lühken et al., 2016; Wiedemar and Drögemüller, 2015; Pickering et al., 2009). Yet there were exceptions from that rule. Heterozygous sheep do not fit the scheme at all as this genotype was found in male and female polled, horned, and scured (except males) sheep. In addition, sheep with oval horns do not fit into the scheme as there was no oval-horned individual without the *RXFP2* insertion. Maybe this horn phenotype is independent from the *RXFP2* variant or, in contrast, is only expressed in individuals with at least one copy of the *RXFP2* insertion. However, to prove this, a larger sample set would be needed. In comparison to the other horn phenotypes, the inheritance of both oval horns and scurs is the least comprehensible. Taken together, an influence of the *RXFP2* insertion on horn status (in terms of the presence or absence of horns or scurs) cannot be ruled out, but this is not seen consistently in Icelandic sheep. Among the breeds analyzed by Lühken et al. (2016), the Bavarian Forest breed showed the greatest similarity with the Icelandic sheep analyzed here in terms of variability of horn status and the *RXFP2* variant. Based on this, it is also not surprising that the SNV OAR10_29458450 close to the above-mentioned insertion, which can be used as a polled-predicting variant in Merino sheep (Duijvesteijn et al., 2018), seems not to be a suitable marker for horn status in Icelandic sheep. Only the wild type was found in the investigated sheep, regardless of their horn status. Taken together, the current findings can be considered to be an indication that more than just one gene locus influences the horn status in sheep, as has also been seen in cattle (scurs: Gehrke et al., 2020; Tetens et al., 2015, polledness: Nicholas and Tammen, 2023a, reviewed by Simon et al., 2022). Based on the evolutionary history of the Icelandic sheep breed, it is very likely that they could carry several different variants influencing horn traits.

**Figure 4:**
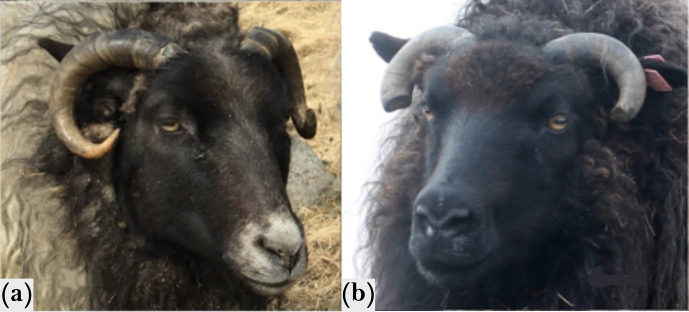
Comparison of two muttons with oval horns originating from farm no. 1. The left sheep **(a)** shows the usual oval horns, while the one on the right-hand side **(b)** shows oval horns that grow sideways towards the face (to avoid injuries, they had to be cut off). The latter was only observed in sheep with OAR10_29461968: CC. Please note that the animals are not exactly the same age; therefore, no comparison of horn size or length should be made.

As information about the horn morphology of the sampled horned sheep was available, we examined a possible association with the previously published haplotype that showed a segregation with horn size and form in Chinese breeds (Pan et al., 2018). These also showed either rather spiral or oval horns, comparable to horn shapes occurring in Icelandic sheep. However, a segregation of the haplotype 2 with a certain horn form was not verified for the tested Icelandic sheep. Surprisingly, none of the analyzed sheep, regardless of the horn phenotype, carried the so-called haplotype 2 (OAR10 29 461 968: T + OAR10 29 462 010: T), which Pan et al. (2018) reported to be common in breeds with curled or spiral horns. In contrast to the sheep used by Pan et al.  (2018), no length measurements were available for the examined Icelandic sheep. However, the breeder reported retrospectively that all the sheep homozygous with regard to the T allele (OAR10_29461968) were the ones that developed the “strongest” horns. Furthermore, as the allele T of SNP OAR10_29461968 was mainly present in normally horned sheep, the previously seen connection of increased horn length with the amount of T copies (Pan et al., 2018) seems also to be observed in Icelandic sheep. Furthermore, the breeder noted that, among the oval-horned sheep, the appearance of the horns of the only two sheep homozygous with regard to C was very similar, while the other sheep with oval horns (with genotypes CT and TT) differed from these two (Fig. 4).

However, without specific horn length measurements at a certain age for the sheep analyzed, the influence of the SNV OAR10_29461968 cannot be evaluated exactly, but tendencies can be pointed out. Interestingly, Sim and Coltman (2019) could not confirm the mentioned association for Thinhorn sheep. In those, none of the loci mentioned before were significantly associated with horn size, but instead, two other SNVs on chromosome 2 and 3 (OAR2_43601714 and OAR3_134140997, respectively) were shown to be associated with horn length (Sim and Coltman, 2019). One problem of studying a quantitative trait, which is as diverse as horns, is the correct phenotyping for classification, especially when it comes to morphology or horn status in breeds with a variable status and the occurrence of scurs. Therefore, it is also possible that, although the horn shapes show great similarities, they are nevertheless different phenotypes. In such a case, it would not be surprising that no association was found in Icelandic sheep as the transferability of the findings from Pan et al. (2018) would be low.

We were able to confirm the association between the *HOXD1* variant (Allais-Bonnet et al., 2021) and the occurrence of multi-hornedness (polyceraty) in the analyzed Icelandic sheep. Only sheep from the multi-horned flock carried the associated *HOXD1* deletion. In addition, no individual from the polycerate family with only two horns carried it. Interestingly, four polled sheep originating from the multi-horned family showed the 4 bp deletion as well. Until now, this has been observed as a dominant trait when compared with two horns, and we expected to observe multi-hornedness in all sheep carrying the *HOXD1* deletion. No comparison with former results can be made as the sheep analyzed by Allais-Bonnet et al. (2021) and partly also by Greyvenstein et al. (2016) were all phenotyped as polycerate or two-horned or scured – no polled individual was mentioned in these studies. Polledness is not reported to occur in Jacob sheep consistently and is just reported for females in the breeds Navajo-Churro and Damara (Porter et al., 2016).

To the best of our knowledge, this is the first study in which polycerate sheep and polled family members were genotyped simultaneously for the 1.78 kb sized *RXFP2* insertion and the *HOXD1* 4 bp deletion. Based on our results in a low number of samples, it seems that polledness in sheep with the *HOXD1* deletion is not caused by the presence of the *RXFP2* insertion. Notably, even a single polycerate polled ewe carried the *RXFP2* wild type. A further investigation of polledness in sheep carrying the *HOXD1* deletion needs to be conducted with a larger sample set in the future. However, as far as can be hypothesized from the present results, it seems that at least one other variant besides the *RXFP2 *insertion controls the absence of horns in polycerate animals. This probably acts epistatically on the *HOXD1* variant, resulting in polled sheep in the presence of the polyceraty allele (4 bp del in *HOXD1*).

A recent study found that genes such as *FOXL2*, *TNN*, and *ACAN*, in addition to the well-known *RXFP2*, are involved in horn development in ovines (Luan et al., 2023). This supports the assumption that other gene variants have an impact on the complex horn phenotype trait. Just recently, a study on whole-genome sequences of more than 1000 sheep (representing ∼150 breeds and seven wild sheep species) revealed three major haplogroups (hap-a, hap-b, hap-c) in the *RXFP2* region, which were highly frequent in polled, sex-specific, and horned breeds, respectively (Cheng et al., 2023). There is evidence that these haplogroups were introgressed from Iranian mouflon. Nevertheless, it is still possible that all direct ancestors of domestic sheep carried them as well (Cheng et al., 2023). Furthermore, it was postulated that at least hap-c was introgressed before the worldwide spread related to the domestication of sheep (Cheng et al., 2023). However, since no further alleles associated with polledness in sheep have been identified in the meantime (Nicholas and Tammen, 2023b), many questions, especially on the breed-specific and sex-dependent genetic control over the presence or absence of horns, remain unanswered.

## 5 Conclusions

As in other sheep breeds with variable horn status, the inheritance of horn status (in terms of presence or absence) proved to be complex in Icelandic sheep, especially when sheep carry anything other than regularly formed horns. However, polled × polled matings seem to be a relatively reliable way to produce polled offspring.

To our knowledge, this is the first detailed study of horn status in Icelandic sheep that also includes polyceraty, as well as horn shape, based on already-known variants and markers. Although nearly all polled Icelandic sheep carried the 1.78 kb sized *RXFP2* insertion, at least on one chromosome, and although the majority of regularly horned seep were homozygous with regard to the *RXFP2* wild type, similarly to other sheep breeds with variable horn status, no perfect segregation of this variant with horn status was observed, especially in sheep with scurs and oval horns.

A trend in association was also observed for the previously published link between the SNP OAR10_29461968 (TT), located in the *RXFP2* gene, and increased horn length in Icelandic sheep.

The interplay of polyceraty, which segregated perfectly with the published 4 bp deletion in *HOXD1 *in Icelandic sheep, and polledness should be investigated in more detail on a larger sample set and by also taking into account other variants besides the 1.78 kb sized *RXFP2* insertion.

As an isolated population with extensive information about the individual animal, the Icelandic sheep provide a promising basis for further investigations considering horn-status-related and other traits, as well as for diversity analyses. Follow-up investigations will be needed for larger sample sets, with more detailed information on horn morphology, and these should also make use of techniques that have been further developed in the meantime, such as long-read sequencing, to address potentially more involved, complex variants.

## Supplement

10.5194/aab-67-237-2024-supplement The supplement related to this article is available online at: https://doi.org/10.5194/aab-67-237-2024-supplement.

## Data Availability

For detailed information on the data, please refer to Table S3.
