# Supplementary material for: Analysis of genetic variants for different horn phenotypes and their inheritance in Icelandic sheep
Source: Arch Anim Breed. 2024 Jun 5;67(2):237–46. doi: 10.5194/aab-67-237-2024 (PMC13159734; doi:10.5194/aab-67-237-2024)
Supplement: The supplement related to this article is available online at: https://doi.org/10.5194/aab-67-237-2024-supplement. [file aab-67-237-2024-supplement.pdf]

Supplement of Arch. Anim. Breed., 67, 237–246, 2024
https://doi.org/10.5194/aab-67-237-2024-supplement
© Author(s) 2024. CC BY 4.0 License.

Open Access
Archives Animal Breeding

Supplement of

Analysis of genetic variants for different horn phenotypes and their inheritance in Icelandic sheep

Rebecca Simon et al.

Correspondence to: Gesine Lühken (gesine.luehken@agrar.uni-giessen.de)

The copyright of individual parts of the supplement might differ from the article licence.

Supplementary material

Table S1 Genotyping results for the previously published SNPs OAR10_29 461 968 and OAR10_29 462 010 that showed an influence on horn length and form in Chinese breeds (Pan et al. 2018) in selected Icelandic sheep with the displayed horn phenotypes. Please note that for the latter SNP just the C allele was present in the analysed sample set.

		OAR10_29 461 968			OAR10_29 462 010
**Horn phenotype**	**Sex**	**C / C**	**T / C**	**T / T**	**C / C**
polled	female	1	1		2
	male				
normal horned	female		1	1	2
	male		3	4	7
	N/A			1	1
scurs	female		6		6
	male		3		3
oval horns	female			1	1
	male	2	9		11
polycerate (4-6 horns)	female			1	1
	male		1	1	2
polled polycerate (4-6 horns)	female		1	1	2
	male		1		1
	N/A		1		1

Table S2 Horn status of the offspring from various matings on farm N°1, indicating which horn status derived from which parent combination. Please note that for some parents no detailed horn information was available, indicated by horns (?).

**Mating of different horn status parents**			**Horn status progeny**			
			**Polled**	**Normal horned**	**Scurs**	**Oval horns**
polled	X	polled	12		2	1
polled	X	scurs	5			3
polled	X	normal horns	2	4		2
polled	X	oval horns	8		4	
normal horns	X	normal horns	1			1
normal horns	X	oval horns		1		
normal horns	X	scurs		2		
oval horns	X	oval horns	1		1	2
oval horns	X	scurs	3			3
polled	X	horns (?)	2			
scurs	X	horns (?)			1	
?	X	polled	1			
?	X	horns (?)			1	1
?	X	scurs		2		
?	X	?	3		1	

Table S3 Details on all Icelandic sheep used for the analysis. Please note that the individuals used for genotyping of the different variants are marked.

farm	lab N°	sex	horn status	details on horn from or number	details on mothers horn phenotypes	details on fathers horn phenotypes	RXFP2 insertion (Wiedemar and Drögemüller (2015))	OAR10_29458450 (Dujivesteijn et al. (2018))	OAR10_29461968 ("Pan5", Pan et al. (2018))	OAR10_29462010 ("Pan6", Pan et al. (2018))	HOXD1 deletion (Allais-Bonnet et al. (2021))
N°1	20224504	female	horns	normal, 2	scurs	unkown	✓	✓	✓	✓	✓
N°1	20213679	female	horns	normal, 2	scurs	horns, normal, 2	✓	✓	✓	✓	✓
N°1	20223748	male	horns	normal, 2	scurs	unkown	✓	✓	✓	✓	✓
N°1	20213677	male	horns	normal, 2	horns, oval	horns, normal, 2	✓	✓	✓	✓	✓
N°1	20223678	male	horns	normal, 2	scurs	horns, normal, 2	✓	✓	✓	✓	
N°1	20213683	male	horns	normal, 2	polled	horns, normal, 2	✓	✓	✓	✓	
N°1	20213682	male	horns	normal, 2	polled	horns, normal, 2	✓	✓	✓	✓	
N°1	20213680	male	horns	normal, 2	polled	horns, normal, 2	✓	✓	✓	✓	
N°1	20213681	male	horns	normal, 2	polled	horns, normal, 2	✓	✓	✓	✓	
N°1	20214213	female	horns	oval	unkown	horns, no details	✓		✓	✓	✓
N°1	20223773	male	horns	oval	polled	polled	✓		✓	✓	✓
N°1	20223749	male	horns	oval	scurs	polled	✓		✓	✓	
N°1	20223764	male	horns	oval	scurs	unkown	✓		✓	✓	
N°1	20223776	male	horns	oval	horns, oval	horns, oval	✓		✓	✓	
N°1	20223771	male	horns	oval	horns, oval	horns, oval	✓		✓	✓	
N°1	20223766	male	horns	oval	scurs	horns, oval	✓		✓	✓	
N°1	20223751	male	horns	oval	scurs	horns, oval	✓		✓	✓	
N°1	20223768	male	horns	oval	scurs	horns, oval	✓		✓	✓	
N°1	20211430	male	horns	oval	polled	horns, normal, 2	✓		✓	✓	
N°1	20211431	male	horns	oval	polled	horns, normal, 2	✓		✓	✓	
N°1	20223745	male	horns	oval	horns, normal, 2	horns, normal, 2	✓	✓	✓	✓	
N°1	deceased*	male	horns	oval	scurs	polled	✓				
N°1	20213950	female	polled		polled	horns, no details	✓	✓			
N°1	20213949	female	polled		scurs	polled	✓	✓			
N°1	20213956	female	polled		unkown	unkown	✓	✓			
N°1	20213955	female	polled		unkown	unkown	✓	✓			
N°1	20223750	female	polled		polled	polled	✓	✓			✓
N°1	20223756	female	polled		scurs	polled	✓	✓			✓
N°1	20213954	female	polled		polled	polled	✓	✓			
N°1	20223757	female	polled		polled	polled	✓	✓			
N°1	20223775	female	polled		polled	polled	✓	✓			
N°1	20224503	female	polled		polled	polled	✓	✓			
N°1	20223760	female	polled		polled	polled	✓	✓			
N°1	20223767	female	polled		scurs	horns, oval	✓	✓			
N°1	20223772	female	polled		polled	horns, oval	✓	✓			
N°1	20223765	female	polled		polled	horns, oval	✓	✓			
N°1	20223774	female	polled		polled	horns, oval	✓	✓			
N°1	20223779	female	polled		polled	horns, oval	✓	✓			
N°1	20223753	female	polled		polled	polled	✓	✓			
N°1	20223763	female	polled		polled	polled	✓	✓			
N°1	20223755	female	polled		scurs	horns, oval	✓	✓			
N°1	20223747	female	polled		scurs	horns, oval	✓	✓			
N°1	20223770	female	polled		horns	polled	✓	✓			
N°1	20213952	female	polled		polled	unkown	✓	✓			

Table S3 Details on all Icelandic sheep used for the analysis. Please note that the individuals used for genotyping of the different variants are marked. (continued)

**farm**	**lab N°**	**sex**	**horn status**	**details on horn from or number**	**details on mothers horn phenotypes**	**details on fathers horn phenotypes**	**RXFP2 insertion (Wiedemar and Drögemüller (2015))**	**OAR10_29458450 (Dujivesteijn et al. (2018))**	**OAR10_29461968 ("Pan5", Pan et al. (2018))**	**OAR10_29462010 ("Pan6", Pan et al. (2018))**	**HOXD1 deletion (Allais-Bonnet et al. (2021))**
N°1	20223762	female	polled		polled	polled	✓	✓			
N°1	20211428	female	polled		polled	horns, normal, 2	✓	✓			
N°1	20211429	female	polled		polled	horns, normal, 2	✓	✓			
N°1	20223746	female	polled		horns, normal, 2	horns, normal, 2	✓	✓	✓	✓	✓
N°1	deceased*	female	polled		horns, oval	polled	✓				
N°1	deceased*	female	polled		horns, oval	polled	✓				
N°1	deceased*	female	polled		scurs	polled	✓				
N°1	deceased*	female	polled		polled	polled	✓				
N°1	deceased*	female	polled		horns, oval	horns, oval	✓				
N°1	deceased*	female	polled		scurs	polled	✓				
N°1	20223758	male	polled		polled	polled	✓	✓			✓
N°1	20223754	male	polled		scurs	polled	✓	✓			✓
N°1	20223777	male	polled		polled	horns, oval	✓	✓			
N°1	20223778	male	polled		polled	horns, oval	✓	✓			
N°1	deceased*	male	polled		polled	polled	✓				
N°1	20221644	male	polled		unkown	unkown	✓	✓			
N°1	20222931	female	scurs		polled	horns, no details	✓		✓	✓	
N°1	20213951	female	scurs		unkown	horns, no details	✓		✓	✓	
N°1	20214317	female	scurs		unkown	unkown	✓		✓	✓	
N°1	20220500	female	scurs		polled	horns	✓		✓	✓	✓
N°1	20223759	female	scurs		polled	horns	✓		✓	✓	
N°1	20223744	female	scurs		scurs	horns, normal, 2	✓	✓	✓	✓	✓
N°1	20223761	male	scurs		horns, oval	horns, oval	✓		✓	✓	✓
N°1	20223769	male	scurs		polled	polled	✓		✓		
N°1	20223752	male	scurs		polled	horns, oval	✓		✓		
N°1	deceased*	male	scurs		polled	polled	✓				
N°2	20223040	male	horns	polycerate origin, unknown			✓				✓
N°2	20223041	female	horns	polycerate origin, 2			✓				✓
N°2	20223050	female	horns	polycerate origin, 2			✓				✓
N°2	20223059	female	horns	polycerate origin, 2			✓				✓
N°2	20223035	male	horns	polycerate origin, 2			✓	✓			✓
N°2	20223036	male	horns	polycerate origin, 2			✓	✓			✓
N°2	20223037	male	horns	polycerate origin, 2			✓				✓
N°2	20223038	male	horns	polycerate origin, 2			✓				✓
N°2	20223039	male	horns	polycerate origin, 2			✓				✓
N°2	20223044	male	horns	polycerate origin, 2			✓				✓
N°2	20223045	male	horns	polycerate origin, 2			✓				✓
N°2	20223046	male	horns	polycerate origin, 2			✓				✓
N°2	20223049	male	horns	polycerate origin, 2			✓				✓
N°2	20223058	male	horns	polycerate origin, 2			✓				✓
N°2	20223062	male	horns	polycerate origin, 2			✓				✓
N°2	20223063	male	horns	polycerate origin, 2			✓	✓			✓
N°2	20223061	unknown	horns	polycerate origin, 2			✓	✓	✓	✓	✓
N°2	20223053	female	horns	polycerate origin, 2			✓	✓			✓

Table S3 Details on all Icelandic sheep used for the analysis. Please note that the individuals used for genotyping of the different variants are marked. (continued)

farm	lab N°	sex	horn status	details on horn from or number	details on mothers horn phenotypes	details on fathers horn phenotypes	RXFP2 insertion (Wiedemar and Drögemüller (2015))	OAR10_29458450 (Dujivesteijn et al. (2018))	OAR10_29461968 ("Pan5", Pan et al. (2018))	OAR10_29462010 ("Pan6", Pan et al. (2018))	HOXD1 deletion (Allais-Bonnet et al. (2021))
N°2	20223054	female	horns	polycerate origin, 2			✓	✓			✓
N°2	20223060	male	horns	polycerate origin, 4			✓	✓	✓	✓	✓
N°2	20221862	unknown	horns	polycerate origin, 4			✓				
N°2	20211413	female	horns	polycerate origin, 6			✓		✓	✓	✓
N°2	20223051	male	horns	polycerate origin, 4			✓	✓	✓	✓	✓
N°2	20223042	female	polled	polycerate origin, 2			✓				✓
N°2	20223043	female	polled	polycerate origin, 2			✓				✓
N°2	20223048	female	polled	polycerate origin, 2			✓				✓
N°2	20223047	male	polled	polycerate origin, 2			✓				✓
N°2	20223055	male	polled	polycerate origin, 2			✓				✓
N°2	20223052	female	polled	polycerate origin, 2			✓	✓	✓	✓	✓
N°2	20223034	female	polled	polycerate origin, 4			✓	✓	✓	✓	✓
N°2	20223057	female	polled	polycerate origin, 4			✓	✓	✓	✓	✓
N°2	20223056	male	polled	polycerate origin, 4			✓	✓	✓	✓	✓
N°2	20223033	unknown	polled	polycerate origin, 4 (scurs?)			✓	✓	✓	✓	✓

* no DNA samples available
